# 3D porosity structure of the earliest solar system material

**DOI:** 10.1038/s41598-022-11976-1

**Published:** 2022-05-19

**Authors:** Romy D. Hanna, Richard A. Ketcham, David R. Edey, Josh O’Connell

**Affiliations:** Jackson School of Geosciences, University of Texas at Austin, Austin, 78712 USA

**Keywords:** Meteoritics, Asteroids, comets and Kuiper belt, Early solar system

## Abstract

Carbonaceous chondrites (CCs) contain the earliest preserved Solar System material, and objects containing this material are targets of numerous sample return missions. Both laboratory and remote sensing data have shown that this material can be highly porous, but the origin and nature of this porosity is currently not well understood. Because the majority of porosity within CCs is submicron to micron in size, previous lab efforts have been restricted by the limited observational scale required to examine this porosity with currently available techniques. Here we present results from a newly developed technique that allows submicron porosity to be examined in 3D within a 12 mm^3^ volume of CM Murchison. We use X-ray computed tomography combined with the highly attenuating noble gas xenon to characterize porosity well below the spatial resolution of the data (3.01 µm/voxel). This method not only allows examination of submicron porosity within a significantly larger volume than previously possible but also reveals the full three-dimensional porosity structure and pore connectivity. Our data reveal that some fine-grained rims (FGRs) surrounding chondrules have a complex 3D porosity structure, suggesting formation of the FGRs via dust aggregates or variable secondary processing around the rim after accretion.

## Introduction

Porosity is an important material property that greatly affects a wide range of physical processes on asteroids. It significantly influences cratering mechanics; not only does it contribute to the attenuation of impact shock waves but it also determines the amount and distribution of waste heat generated^1–3^. Porosity affects permeability and the movement of gases and fluids through an object, controlling the extent and type (e.g., open- vs. closed- system) of aqueous alteration e.g., ^4^. It also influences a material’s thermal conductivity and therefore its thermal inertia^5^, which has implications for the movement of heat and energy within an asteroid. Porosity also has also been shown to have a significant effect on the outcome of kinetic impacts, as might be used to deflect Near Earth Asteroids (NEAs)^6^. Therefore, determination of the porosity, as well as its distribution and structure, has significant implications for the physical, hydrological, and dynamical evolution of an asteroid.

Recently, two sample return missions to carbonaceous asteroids (162,173) Ryugu and (101,955) Bennu, by JAXA’s Hayabusa2 and NASA’s Origins, Spectral Interpretation, Resource Identification, and Security–Regolith Explorer (OSIRIS-REx) missions, respectively, have revealed that the asteroids are significantly more microporous than expected^7,8^, and the preliminary analysis of samples from Ryugu confirm that they are more highly microporous (46%) than their analog CI meteorites^9^. Microporosity in this context is defined as porosity on the scale of the analog meteorite, and thus is composed of inter- and intragrain porosity and small fractures (hundreds of microns in width or less). The origin of this unexpectedly high porosity is not known. It could represent porosity created from a secondary process such as meteoroid bombardment or cracking due to diurnal thermal stress^8^. However, it is hypothesized that it is more likely the original, primary porosity of the carbonaceous chondrite material that accreted to the asteroid^7,8^.

The porosity of carbonaceous chondrites (CCs) have been previously measured in the lab using both bulk (He pycnometry) and direct imaging (scanning or transmission electron imaging (SEM/TEM); X-ray computed tomography (XCT)) methods e.g., ^10–13^. These studies have shown that the CC types that are the closest analogs to carbonaceous asteroids Bennu and Ryugu, the CMs, CIs, and ungrouped C2s, have a high porosity (~ 23–40%) that is primarily composed of submicron to micron sized pores^10–12,14–17^. While bulk porosity measurements are likely to be accurate, they lack any detail on the type (intragranular, intergranular, fracture, etc.), morphology, or location of the porosity. Direct imaging of the pores provides this detail, but 2D imaging methods such as SEM or TEM requires destructive preparation (sectioning) of the sample. It also examines only a limited area (for TEM, on the order of ~ 100 µm^2^), which may not be representative of the sample, and does not provide 3D context, which we will show can be critical in the interpretation of the origin and evolution of the porosity. Further, knowledge of the 3D porosity distribution within a sample is important for studies of carbonaceous chondrites that employ freeze–thaw disaggregation to concentrate components of interest such as chondrules, refractory inclusions, presolar grains, or clasts e.g., ^18^.

XCT is able to examine porosity within larger, more representative samples while preserving 3D spatial context. XCT is a nondestructive imaging technique that produces a series of two-dimensional (2D) images (slices) where the gray scales in each image represent X-ray attenuation, which is to first order dependent on the density and atomic number (Z) of the material^19^. A few studies have used XCT to examine porosity within chondrites but have been limited by the scale of observation due to only attempting to identify discrete pores^13,20,21^. Typically, a discrete 3D feature (such as a pore) can be accurately measured only when it has a diameter of at least a few (~ 3) voxels^19,22^. Consequentially, when imaging small (6–12 mm^3^) chondrite chips, Friedrich and Rivers ^20^ found that they could not measure all the porosity within highly porous samples (> 15%) due to the large number of pores below the scanning resolution (2.6 µm/voxel). Conversely, Dionnet, et al. ^13^ used a very high imaging resolution (0.13 µm/voxel) for CM Paris but also found a porosity much lower than expected (4.6%) compared to previous estimates (30%^15^), most likely due to the unrepresentativeness of their tiny sample (40 µm in diameter).

However, XCT imaging with a heavy noble gas such as Kr or Xe, which is highly attenuating to X-rays, has allowed inspection of extremely fine-scale porosity in terrestrial samples^23–25^. By XCT imaging a porous sample twice, once in air or under vacuum and once infiltrated by the gas, and then subtracting the former from the latter, one obtains a 3D map of where the gas has infiltrated, and thus the connected porosity. In such maps, each voxel value corresponds to partial porosity, or the fraction of the voxel that contains pore space, thus revealing the location of all interconnected porosity, at all scales. The noble gas technique has two other advantages over direct XCT pore imaging. First, it can differentiate between low attenuation material, such as organics, and a nano-porous region with porosity below the spatial resolution, which is otherwise indistinguishable from a low attenuation material. Second, it provides information on the connectivity of the pores, as isolated pores will not change in X-ray attenuation with the introduction of the gas. In this work, we apply this XCT noble gas imaging method for the first time to an extraterrestrial sample, CM Murchison (an analog meteorite for carbonaceous asteroids), to demonstrate and refine the technique for application to current and future sample return missions to these highly microporous and complex targets.

## Results

**Figure 1:**
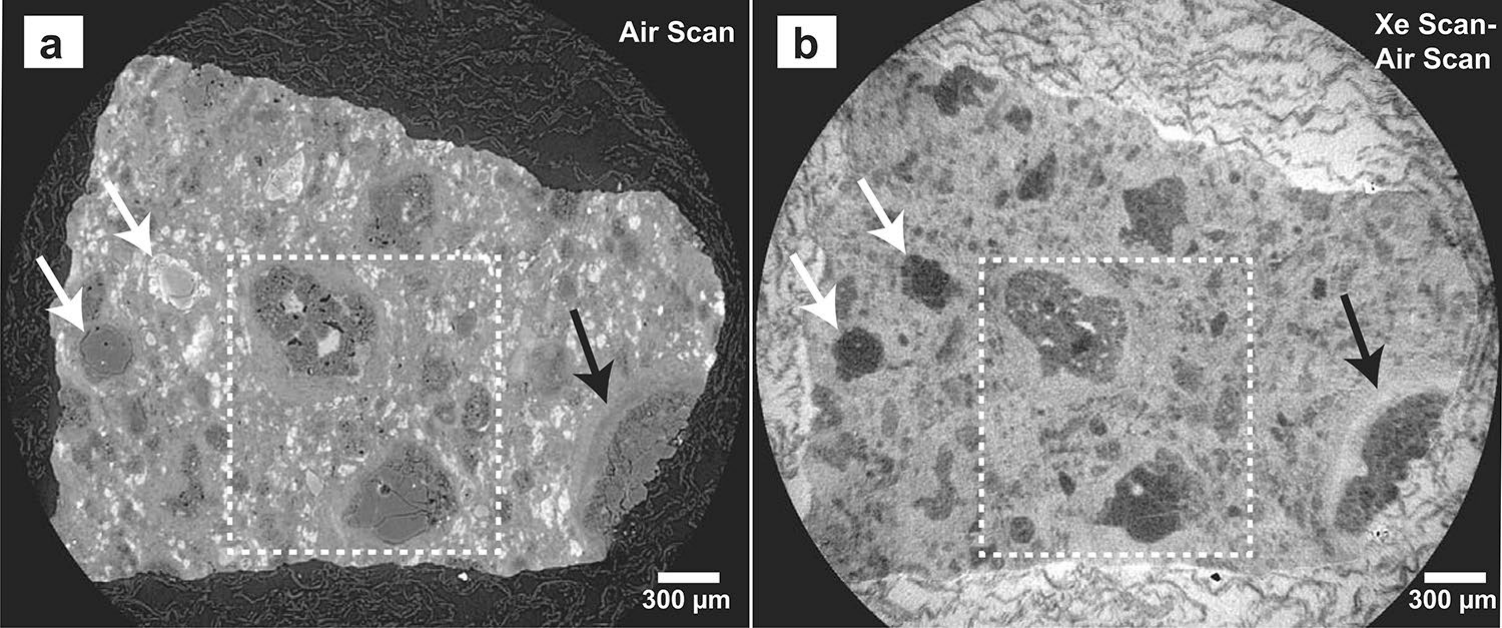
XCT Slice 420. (**a**) Air scan (**b**) Xe scan—Air scan (i.e., relative amount of infiltrated Xe). In (**b**) the darkest areas represent no Xe penetration, the lightest areas show where Xe has penetrated. The highest amount of Xe is present within the space surrounding the sample; the structure visible is that of the Kimwipe™ which surrounded the sample. The matrix shows a relatively higher proportion of Xe infiltration compared to the chondrules. White arrows mark two chondrules with significantly different X-ray attenuation (a) that both show relatively low Xe penetration (i.e., are non-porous) (b), confirming that the subtraction dataset in (b) shows only Xe infiltration (i.e., relative porosity) and not the X-ray attenuation of the materials. Black arrow highlights layering of relative xenon penetration within the rim of a chondrule. White dashed box shows location of Fig. 2.

We imaged a 50 mg chip (~ 3 mm in size) of CM Murchison with XCT twice—once in the presence of pressurized xenon gas (2.76 MPa) and once in atmospheric air. We aligned, scaled, and subtracted the two XCT data volumes to derive a 3D volume of relative xenon infiltration. As expected the highest amount of xenon was found in the space surrounding the samples (Fig. 1), but a significant amount of xenon also infiltrated the sample interior indicating a high degree of interconnected porosity that is below the scale of the CT data (3.01 µm/voxel). Chondrules and sulfides show significantly lower xenon infiltration compared to the surrounding matrix and fine-grained rims (FGRs). Some FGRs show a higher degree of xenon infiltration compared to the nearby matrix, as well as evidence of layering within the FGR itself (Fig. 1b). This layering is evident in the XCT scan of the sample in air (Fig. 1a). Because X-ray attenuation is a strong function of composition, this FGR layering within the air scan could be interpreted as compositional changes within the layer, similar to olivine chondrules that have different X-ray attenuations due to different their different olivine compositions^26^ (Fig. 1a). However, the xenon infiltration image reveals that while the olivine chondrules have a very similar, low abundance of xenon infiltration despite their large relative X-ray attenuations, the layering within the FGR is due to a difference in xenon infiltration, and therefore porosity, within the rim (Fig. 1b). This porosity is sub-voxel in size (< 3 µm), and therefore it is the presence of this porosity, rather than a different chemical composition, that is lowering the X-ray attenuation with the voxel. Sub-voxel porosity causes a common XCT imaging artifact referred to as the partial volume effect (PVE)^27^, and a strength of the noble gas infiltration technique used here is that it can uniquely determine whether a lowered X-ray attenuation within a single voxel is due to porosity (i.e., PVE) or chemical composition.

**Figure 2:**
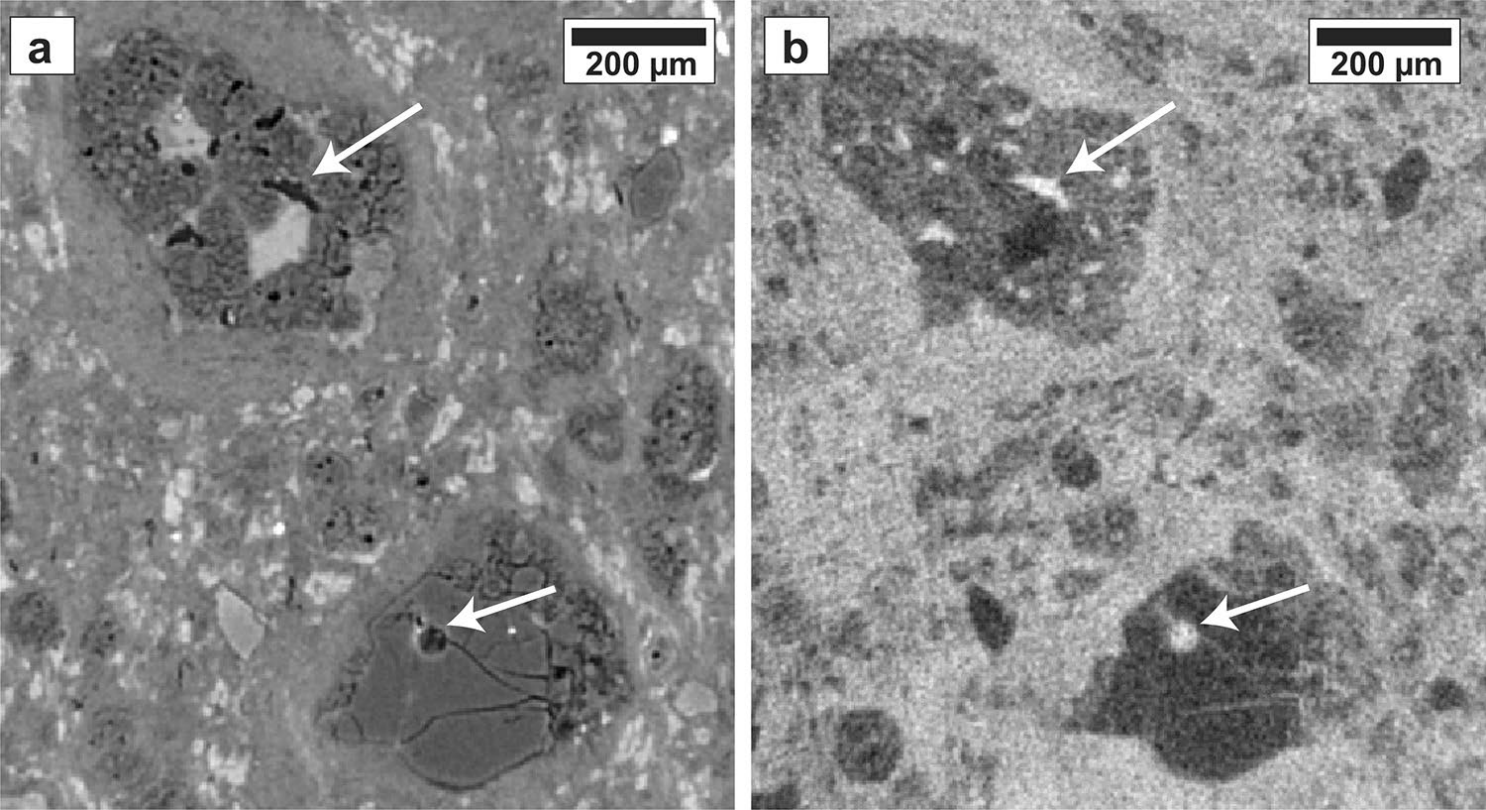
Detail of slice 420 shown in Fig. 1. (**a**) Air scan (**b**) Xe scan—Air scan (i.e., relative amount of infiltrated xenon). White arrows indicate large interior chondrule pores that infilled with xenon and are therefore not isolated. Their mottled appearance in (**a**) also indicate that they are partially filled with a low-density material. The largest, circular pore (bottom white arrow) measures approximately 40 µm across. The xenon infiltration image (**b**) also highlights fine fractures within the bottom chondrule and areas of lower xenon penetration (i.e., lower porosity) within the top chondrule.

**Figure 3:**
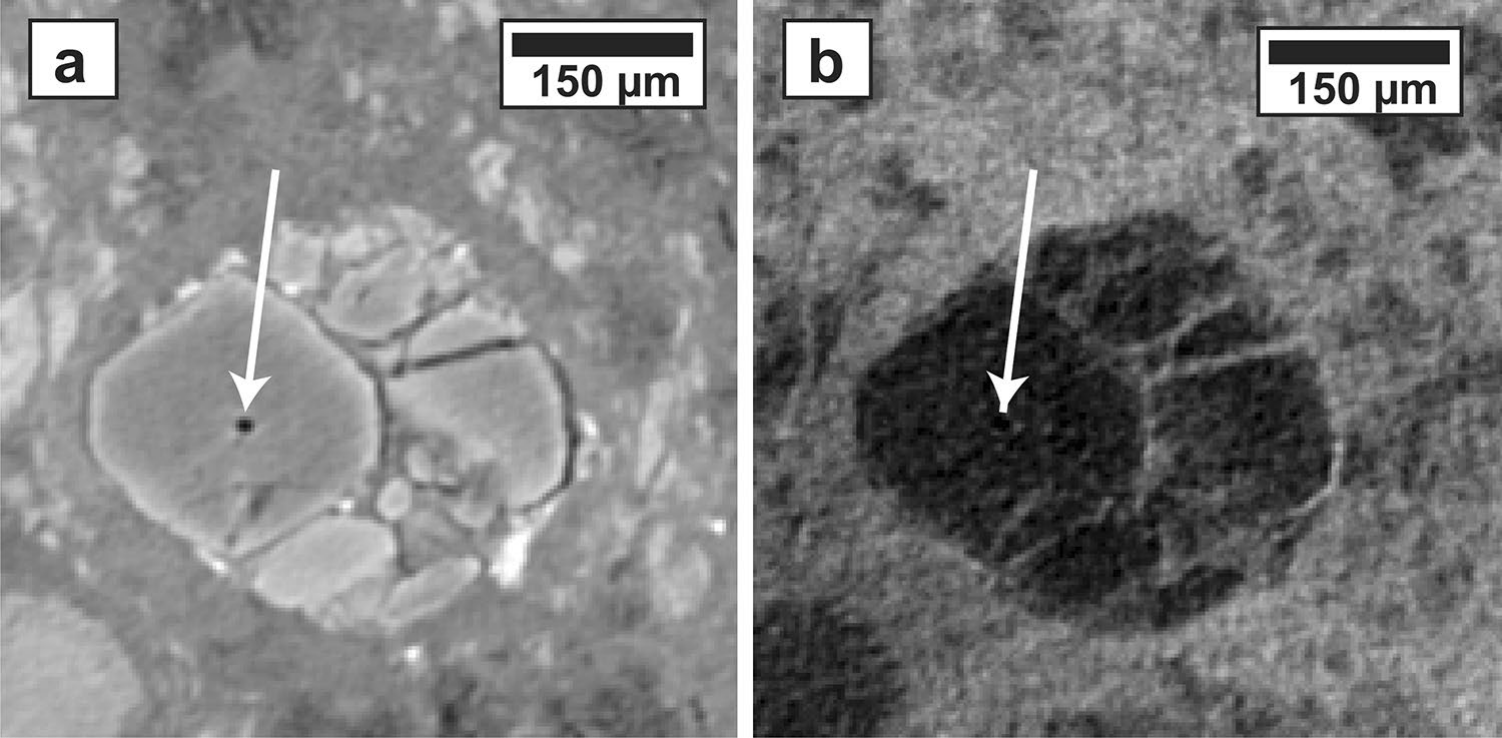
(**a**) XCT slice 316 (magnified by 2x) of Air scan showing an isolated pore (the largest of 5 visible in this crystal) in an Fe-bearing olivine chondrule that has not been infiltrated with xenon (**b**).

Also visible in the xenon infiltration image are small areas of high infiltration within chondrules due to pore space that is visible in the air scan, primarily in the large central chondrule (Fig. 2). The largest of these pores measures approximately 40 µm in its largest dimension, but is also partially filled, as evidenced by both higher attenuation material present in the pore in the air scan (Fig. 2a) and a variation in xenon infiltration across the pore (Fig. 2b). By combining a pore map derived from the Air scan with the xenon infiltration image, we also identified closed porosity within five olivine chondrules, one of which is shown in Fig. 3. Because the sample and chamber were vacuum pumped prior to xenon infiltration and allowed to sit for 30 min to ensure penetration throughout the sample, we are confident these areas are completely closed and isolated. In addition, they are located within individual, euhedral olivines that have a solid crystalline structure with no connecting fractures to the pore.

**Figure 4:**
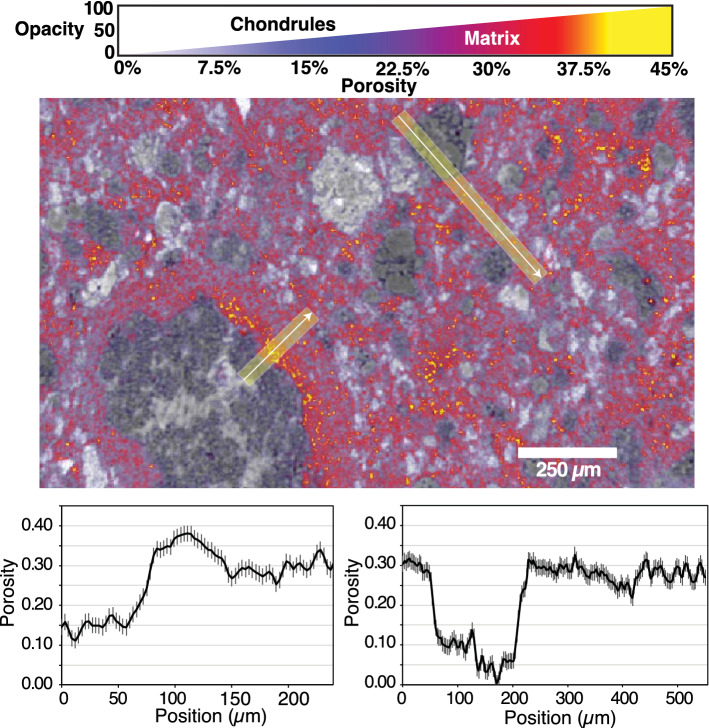
XCT Slice 706 of porosity volume. (Top) Porosity data (colorized with variable opacity) overlaid on XCT data of Air scan (greyscale) showing the distribution of porosity. Porosity value indicated is that inside an individual voxel. White lines with yellow shaded regions show location of line profile plots below. (Bottom) Line profile plots of porosity using 15-pixel wide average (yellow regions on lines in top image) with vertical bars representing the standard error of the mean (2.1%). The matrix has a relatively higher porosity (22–34%) compared to the chondrules (< 17%), and some FGR areas have even higher porosity (up to 38%) than the matrix. See Supplemental Material for animation of full porosity dataset.

**Figure 5:**
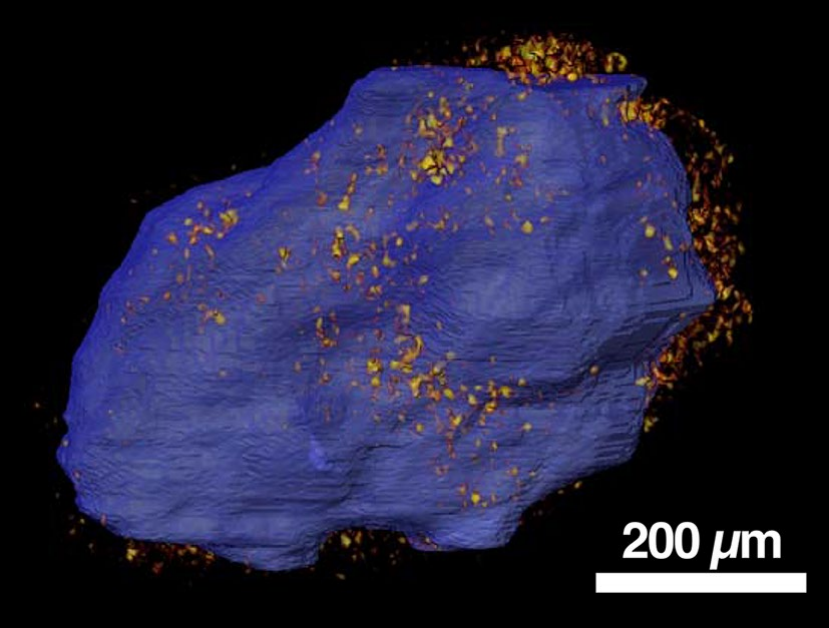
Three-dimensional rendering of chondrule surface (blue) and areas of highest porosity (> 35%) (orange to yellow; same color scale as in Fig. 4) within the FGR. Highest porosity within FGR is localized on the ‘right’ side of the chondrule, and mostly on the ‘top’. The highest porosity regions within the FGR are not consistent layers around the chondrule but isolated patches with a complex geometry. See Supplemental Material for 3D animation.

We next converted an interior subvolume of the Xe infiltration data into porosity values by using the bulk porosity of Murchison measured with He pycnometry (21.9 ± 2.2%) by Macke, et al. ^11^. The porosity of individual voxels in the sub volume ranges from 0 to 56.0% (± 8.2%) porosity and we verify that the matrix has a significantly higher porosity (22–34%) compared to chondrules (< 17%) (Fig. 4). In addition, some FGR areas have even higher porosity (up to 38%) than the matrix, although these high-porosity FGR areas are isolated and do not form a continuous layer around the chondrule (Fig. 4 and 5).

## Discussion

Overall, our porosity results confirm earlier studies that have examined CC porosity. First, we find that the matrix in CM Murchison is highly microporous (22–34%) and is the dominant source of porosity within the sample (Fig. 1). The only other study to quantify the matrix porosity of a CM chondrite (CM Paris, using energy dispersive spectroscopy (EDS)) reported a value of 30%, consistent with our results^15^. Second, we find that some FGRs have a higher porosity (up to 38%) compared to the surrounding matrix, and have a complex porosity structure that can appear as layers in 2D (Fig. 4). Layers of porosity variation within FGRs have also been found in CM Paris, with some layers of the rim showing a higher porosity than the matrix^14^; see their Fig. 4, like those seen in CM Murchison. However, unlike the Zanetta, et al. ^14^ study that was done in 2D, we show that these layers are in fact isolated patches within the rim with a complex 3D geometry (Fig. 5). Therefore, the porosity variation may not be due to changing nebular conditions during multiple, episodic dust deposition events that resulted in continuous layers of varied dust porosities, as suggested previously. They may instead represent patches of dust aggregates with different porosities that were accreted to the rim, as dust aggregates have recently been proposed as an efficient mechanism for building FGRs^28^. These varied dust aggregates could still have experienced different thermal and dynamic compaction histories that resulted in their different porosities, as proposed by the Zanetta, et al. ^14^ model, but prior to, or during, their accretion to the chondrule surface. Another possibility is that secondary processing on the parent, such as impact compaction or aqueous alteration, modified the porosity of the FGRs heterogeneously, erasing any original continuous layering structure. Notably, our previous work has shown that the FGRs are thinner in the direction of the impact force which flattened and foliated the chondrules in this sample^29^, and future work will determine whether the porosity varies around the rim in a way that is consistent with porosity loss due to impact compaction.

A few other studies have noted a porosity difference between FGRs and nearby matrix in CM or CM-like samples, but found that the FGR has a lower porosity than the matrix^10,30^. A lower porosity for some FGRs compared to matrix has also been found in CO DOM 08006^12^. We searched for but did not find any FGRs in CM Murchison that had a significantly lower porosity compared to nearby matrix. This lack of lower relative porosity FGRs could be due to either lower temperatures or less compaction during FGR (or pre-FGR dust aggregate) formation which are hypothesized to result in higher porosity rims^12,14^, or a relatively higher degree of aqueous alteration for CM Murchison on its parent body, which could have lowered the porosity of the matrix relative the FGRs^15^.

Another advantage of combining XCT with noble gas is that pore connectivity can be assessed and visualized. Within this CM Murchison chip we have identified completely closed, isolated pores within five chondrule olivines and were also able to quantify this closed porosity (4.3e-5% of analyzed sample volume) that, although minor in this case, is porosity that cannot be measured with He pycnometry. Because these olivine pores are closed, they are not pores formed through later dissolution of melt inclusions or reactive phases within polycrystalline chondrules on the parent body^31^ or those created via impact shock^21^. Rather, they are primary pores associated with chondrule crystallization in the nebula, most likely vapor or shrinkage bubbles associated with melt inclusions^32,33^ that have been previously identified in Murchison mineral separates using transmitted light^34^. Here, however, we can provide the full 3D context for these pores, and verify that they have been undisturbed since formation, regardless of the extent of impact deformation or aqueous alteration on the CM parent body.

While the noble gas XCT technique detects porosity below the scanning resolution of the data (3.01 µm/voxel, in this study), it cannot distinguish whether the porosity within a voxel is from a single large pore, or multiple smaller ones. Using the previously measured bulk CM Murchison porosity (our sample was too small to measure the bulk porosity directly with He pycnometry), we find that the maximum porosity of a single voxel is 56%. This could signify a single (cubic) pore about 2.5 µm across or multiple pores that are much smaller than this. The smallest voxel porosity we can reliably detect is 8.2% porosity (this is the per-voxel error on our porosity volume), which corresponds to a single pore size of 1.3 µm. However, we know from previous work utilizing 2D scanning electron and transmission electron imaging that the vast majority of pores within carbonaceous chondrites are < 1 µm and down to a few nm in size^10,12,14–16^. Therefore, our technique is most certainly detecting multiple, submicron pores within each voxel. Lastly, if the bulk porosity of our sample is outside the range of that measured for other Murchison samples (21.9 ± 2.2%; Macke et al. ^11^), only the range of absolute porosity values would change. The relative porosity variations that we see among chondrules, matrix and FGRs, and regions within them in Murchison would hold and therefore our results would remain the same.

## Conclusions

The noble gas XCT technique presented herein is the first to measure the 3D submicron porosity, including definitive identification and quantification of closed porosity, of an intact carbonaceous chondrite sample nearly 3 mm in size. It reveals that some fine-grained rims (FGRs) that surround chondrules have a complex 3D porosity structure, suggesting formation of the FGRs via dust aggregates or variable secondary processing around the rim after accretion. This illustrates that the 3D context of submicron pore structure and connectivity are critical to the proper interpretation of porosity formation and evolution, and therefore noble gas XCT will be an essential analytical tool for sample return missions to carbonaceous targets where sample preservation and context is of utmost importance.

## Methods

We imaged a 50 mg chip of CM Murchison (from sample USNM 5487 on loan from the Smithsonian). This is the same Chip A from Hanna and Ketcham^29^ that has been trimmed down in its shortest dimensions to fit inside a 3 mm tube. It is approximately 1 cm in length and the top ~ 3 mm was imaged for this study. The sample was scanned in a 12.7 mm diameter polyetheretherketone (PEEK) rod that was bored out to a 3 mm inner diameter. PEEK is a thermoplastic rated for high pressure work that also has a relatively low X-ray attenuation. A Kimwipe™ was wrapped around the chip to secure it within the tube and limit movement during gas and pressure changes and scanning. The sample chamber was then connected to a series of aluminum tubes and needle valves to a vacuum pump and Xe gas canister with regulator (Supplementary Fig. S1 online). All connections used SwageLok™ connectors and were tested for leaks using N_2_ and an empty chamber before use with the sample.

XCT scans were done at the University of Texas High-Resolution X-ray Computed Tomography Facility (UTCT) on a Zeiss Versa 620 XRM at 90 kV and 12 W with the 0.4X detector, 1601 views, and 2 frames per view. The first scan with Xe gas present in the chamber had a 15 s acquisition time per frame but the air scan (after Xe gas was evacuated from chamber) had only a 10 s acquisition time per frame to avoid saturation of the detector. After loading the sample into the chamber we first pumped the system to low vacuum and then filled the chamber with Xe gas at 2.76 MPa (400 PSI). We also tried scanning at a Xe pressure of 150 PSI but found that this did not provide enough of an X-ray attenuation contrast compared to air to illuminate the porosity within the sample. After the sample chamber was filled with Xe and allowed to permeate the sample for 30 min, we did the first scan that took approximately 15 h, at the conditions listed above. After this first scan (Xe) we slowly opened the needle valve to gradually release the pressurized Xe gas and then let the system sit for 30 min to allow it to equilibrate with ambient air. Then a second scan (Air) was done and took 10.4 h. A beam hardening correction was applied to both scans (0.08 for the Xe scan, 0.5 for the Air scan) using the Zeiss Reconstructer Scout-And-Scan 16.0 software and were reconstructed with a voxel size of 3.01 µm/voxel and exported as series of 32 bit TIFF images. We next aligned the two scan volumes by deleting the last slice (in Z) from the Air scan and shifting it by -1 pixel in the Y direction using ImageJ.

We next rescaled the Air scan volume so non-porous phases had a similar CT value to that of the Xe scan. This is necessary because the polychromatic X-rays are filtered by the Xe gas surrounding the sample, thus exposing it to a different X-ray spectrum compared to that of the Air scan. Further, the different beam hardening corrections for each scan also resulted in a different CT value scaling for each volume. To determine the scaling factor, we measured the mean CT value of 35 phases that represented areas where no Xe penetration was possible, either due to its massive, crystalline form with zero evidence for fractures (olivine, sulfide, metal) or due to it being a pore, or very low attenuation material, that is completely isolated within a crystalline olivine (Supplementary Fig. S2 online; see also Fig. 3). These phases comprised a wide a range of compositions (i.e., X-ray attenuations) and were measured within in each scan (Xe and Air) and plotted. The data was very will fit with a line (R^2^ = 0.996) and so the Air scan was linearly rescaled using this relationship (Supplementary Fig. S2 online). This good fit among all data points also verified that the measured phases did not contain xenon, which would have raised their attenuation in the xenon scan significantly compared to the Air scan. After rescaling, the Air scan volume was subtracted from the Xe scan volume to derive the final CT data volume.

To identify and measure the closed porosity, we made a pore map from the Air scan data (converted to 8bit) using Blob3D^35^ to segment out pores that were a minimum of 3 voxels large and had a CT value of 65 or less. We created a similar map for zero Xe infiltration areas from the Xe-Air CT data volume (converted to 8bit) with Blob3D using a max CT value threshold of 140 and filled holes with a 1 voxel filter width. In this case a minimum voxel size threshold of 9 contiguous voxels was used to more accurately identify truly Xe-free regions within this derived dataset. We next cropped the two maps (pore space and zero Xe infiltration) to a 5.3 mm^3^ interior sub volume of the sample (to avoid beam hardening artifacts around the edges of the sample) and used a logical AND operation to mark voxels that were identified as both pore space and zero Xe infiltration. Only pores that were a minimum of 3 voxels and free from areas of artifacts were considered valid detections of closed pores. Seven separate pores within five olivine chondrules were found with a combined volume of 2.3e-6 mm^3^, representing 4.3e-5% closed porosity within the analyzed sub volume.

We next scaled the CT data volume, which was comprised of unitless CT data values representing effective relative attenuation, into porosity space. This conversion assumes a linear relationship between attenuation of the X-ray signal and the amount of partial porosity (i.e., xenon) within a single voxel, which previous studies have found to be a reasonable approximation^25,36,37^. The surrounding air could not be used as a 100% porosity standard because porosity within the sample will have a different attenuation due to filtering of the polychromatic X-ray spectrum by the sample itself, as well as its variation due to beam hardening (visible as apparent variation in Xe infiltration in air close to the sample in Fig. 1b); the beam hardening correction coefficients were chosen to minimize variation in the solid. The spatial resolution of the scan (3.01 µm/voxel) coupled with measured blur of the scan data of 2.41 ± 1.23 voxels (measured with the method described in^22^) meant that an empty pore or fracture within the chip would have to be a minimum of 24 µm (~ 8 voxels) wide in it shortest dimension to be assured to contain a voxel value representing 100% porosity. We found no features in our chip that were simultaneously wide enough and free of mottling suggesting partial infilling, and so scaled the CT data volume such that the bulk porosity of the chip matched that of Murchison measured with He pycnometry. Because the small size of the chip precluded measurement of its bulk porosity directly with He pycnometry, we used the average bulk porosity (21.9 ± 2.2%) among 14 Murchison samples previously measured by Macke, et al. ^11^. We first cropped the data volume to only the interior of the sample to avoid any remnant beam hardening around the edges of the sample and ensure that the linear relationship between X-ray attenuation and amount of partial porosity holds^36^. We then calculated the average CT value of the 35 Xe-free regions, subtracted this value from the dataset (ensuring zero porosity standards are zero), and scaled the rest of the CT values linearly to a maximum that resulted in a bulk volume porosity of 21.9%. This resulted in a maximum voxel porosity within the sample of 56.0%. To calculate the per-voxel porosity error we did error propagation on the function used to convert the CT data values to porosity and used the standard deviation of the 35 Xe-free regions as the error on the CT data value (this represents the error due to noise and CT artifacts such as beam hardening) and the standard deviation of the measured Murchison porosities (2.2%) as the error (i.e., possible sample heterogeneity) on bulk Murchison porosity. The per-voxel porosity error within the interior sample volume was then calculated and had a mean of 8.2% (7.9–9.7%), but averaged porosity values from multiple voxels (Fig. 4) will be correspondingly lower.

## Supplementary Information

Supplementary Information 1.

Supplementary Video 1.

Supplementary Video 2.

## Data Availability

The two XCT datasets of the USNM 5487 chip (in air and xenon) are archived in the Texas Data Repository and available for download at https://doi.org/10.18738/T8/XYW1BW.
