# Supplementary material for: 3D porosity structure of the earliest solar system material
Source: Sci Rep. 2022 May 19;12:8369. doi: 10.1038/s41598-022-11976-1 (PMC9120439; doi:10.1038/s41598-022-11976-1)
Supplement: Supplementary file 1 — Supplementary Information 1. [file 41598_2022_11976_MOESM1_ESM.pdf]

Supplemental Material for

3D Porosity Structure of the Earliest Solar System Material

Romy D. Hanna*[1], Richard A. Ketcham[1], David R. Edey[1], Josh O'Connell[1]

*corresponding author; romy@jsg.utexas.edu
[1]Jackson School of Geosciences, University of Texas at Austin, 78712

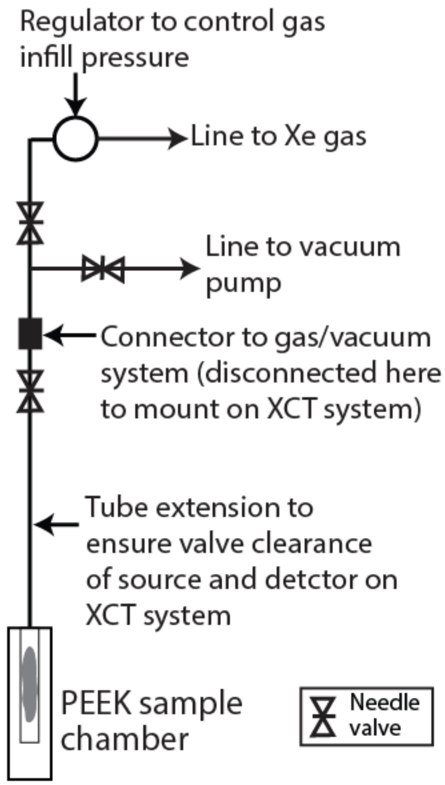

Fig. S1. XCT sample chamber and rig design schematic.

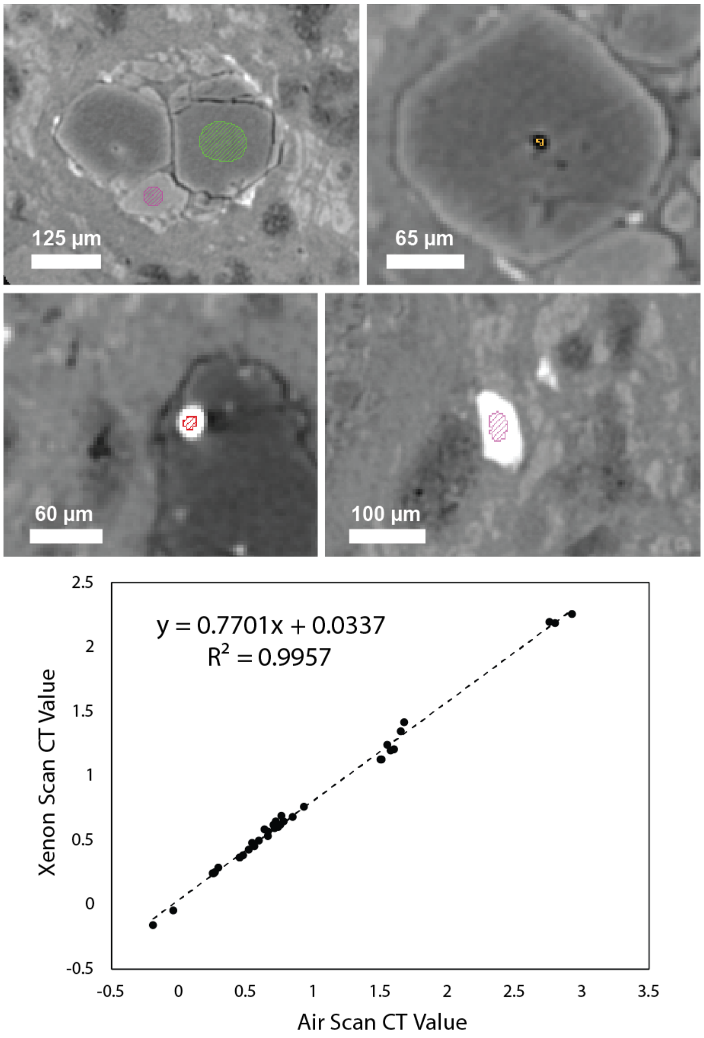

Fig. S2. (Top) Four examples of the phases (olivine, upper left; isolated pore/inclusion, upper right; metal, lower left; sulfide, lower right) used to rescale the Air scan data to match that of the Xe scan. (Bottom) The linear fit to the two scan CT values which was used to linearly scale the Air scan to that of the Xe scan.

PorosityMovie.mp4. Full 3D porosity dataset with same color/opacity scale shown in main text Fig. 4. Width of sample area shown is approximately 1.7 mm.

ChondruleFGRPorosity3D.mp4. Three-dimensional rendering of chondrule surface (blue) and areas of highest porosity (> 35%) (orange to yellow; same color scale as in main text Fig. 4) within the FGR.
